# Safety and tolerability of deferasirox in pediatric hematopoietic stem cell transplant recipients: one facility's five years’ experience of chelation treatment

**DOI:** 10.18632/oncotarget.18725

**Published:** 2017-06-28

**Authors:** Natalia Maximova, Massimo Gregori, Roberto Simeone, Aurelio Sonzogni, Giulia Boz, Carmen Fucile, Valeria Marini, Antonietta Martelli, Francesca Mattioli

**Affiliations:** ^1^ Bone Marrow Transplant Unit, Institute for Maternal and Child Health - IRCCS Burlo Garofolo, Trieste, Italy; ^2^ Department of Pediatric Radiology, Institute for Maternal and Child Health - IRCCS Burlo Garofolo, Trieste, Italy; ^3^ University of Trieste, Trieste, Italy; ^4^ Department of Pathology, Ospedale Beato Papa Giovanni XIII, Bergamo, Italy; ^5^ University of Cagliari, Faculty of Medicine, Cagliari, Italy; ^6^ Pharmacology and Toxicology Unit, University of Genoa, Genoa, Italy

**Keywords:** deferasirox, allogeneic hematopoietic stem cell transplantation (allo-HSCT), pediatric, ductopenia, therapeutic drug monitoring

## Abstract

42 pediatric patients with iron overload, who underwent liver biopsy and DFX treatment after hematopoietic stem cell transplantation were included in the study group. The patients were divided into two groups diversified according to deferasirox trough plasma concentrations (DFX C_trough_) with cut-off equal to10 mcg/mL. The average dose of DFX was 25.9 mg/kg in the DFX C_trough_ < 10 mcg/mL group versus 19.2 mg/kg in the DFX C_trough_ > 10 mcg/mL group (*p*=0,0003). The mean duration of DFX treatment was 135.7 days in the DFX C_trough_ < 10 mcg/mL group versus 41.8 days in the DFX C_trough_ > 10 mcg/mL group (*p*<0.0001). The mean tissue iron concentration in the DFX C_trough_ < 10 mcg/mL group was 261.9 μmol/g versus 133.4 μmol/g in the DFX C_trough_ > 10 mcg/mL group (*p* < 0.0001). 21 patients (100%) in the DFX C_trough_ > 10 mcg/mL group had ductopenia which was complete in 47.6% of them and severe in 52.4%. All patients with particularly high C_trough_ (> 25 mcg/mL) were found to have total ductopenia. 90.5% of all deferasirox-related adverse events and 100% of major adverse events occurred in the DFX C_trough_ > 10 mcg/mL group. In the DFX C_trough_ < 10 mcg/mL group only one patient interrupted chelation therapy versus 16 (84.2%) patients in the DFX C_trough_ > 10 mcg/mL group. We would recommend a close monitoring in pediatric hematopoietic transplant recipients subjected to deferasirox-based therapy because we have observed a high incidence of adverse events and discontinuation of chelation treatment.

## INTRODUCTION

Over recent years, allogeneic hematopoietic stem cell transplantation (allo-HSCT) has become an important technique for treating pediatric diseases, especially hematological and oncological disorders and congenital errors. Iron overload (IO), a relatively common but often neglected transplant-related complication, has been associated with poor prognosis in patients undergoing allo-HSCT for onco-hematological diseases [1].

Iron is an essential element in humans and its quantity is tightly regulated physiologically; however, because humans lack a mechanism for excretion of iron excess, it can be deposited in end organs, leading to severe dysfunction [2]. Transfused red blood cells (RBCs), both during initial treatment and during the post-transplant period, is considered the main cause of iron overload in HSCT recipients [3]. However, IO in patients with hematological malignancies is multifactorial and not exclusively attributable to the intense transfusion regimen. Intensive cytotoxic therapy before HSCT destroys both bone marrow and neoplastic cells, releasing intracellular iron and consequently increasing free iron concentrations [4]. Furthermore, high dose chemotherapy or total body irradiation, components of conditioning prior to HSCT, can damage hepatic cells, resulting in release of intracellular iron pools and contributing to further increases in iron load [5].

Some studies involving pediatric patients have demonstrated that iron-chelating therapy before allo-HSCT significantly reduces transplant-related mortality (TRM) and increases event-free (EFS) and overall survival (OS) [6]. Given that childhood HSCT survivors have longer life expectancies than adult HSCT recipients, they are likely at higher risk of developing long-term complications such as hepatic fibrosis, and cardiac and endocrine dysfunction. Furthermore, earlier large epidemiologic studies have shown that IO increases to risk of oncological diseases [7, 8].

There are few studies regarding the effects of chelation therapy in the post-transplant period in pediatric setting. Additionally, there is a lack of safety and tolerability data about the oral iron chelator deferasirox (DFX) in pediatric patients who have undergone allo-HSCT. The largest study that compared the efficacy and safety of once-daily DFX with phlebotomy had 12 and 14 patients, respectively, in these groups [9].

Our retrospective study of one facility's five years' experience of DFX iron chelation in 42 pediatric patients who underwent allo-HSCT shows that this oral chelator must be carefully used in this setting, including implementing therapeutic drug monitoring (TDM).

## RESULTS

Clinical data of the 42 study patients who had undergone oral chelation therapy with DFX in our Institute were retrospectively reviewed and analyzed. Their age range was 2–17 years.

Most patients (59.5%) had acute lymphoblastic leukemia (ALL). All study patients had undergone allo-HSCT preceded by a myeloablative conditioning regimen.

During the pre-transplantation work-up all patients had undergone an abdominal MRI-based evaluation of iron concentration in the liver, pancreas, spleen, and bone. All patients were found to have IO, which was assessed as moderate in 16 patients (38.1%) and severe in 26 patients (61.9%). The mean number of analyzed organs affected by iron overload was: 3.1 (range: 2–4).

Moreover, all patients had undergone a liver biopsy before leaving the Bone Marrow Transplant Unit. Histological examination demonstrated the presence of ductopenia in 39 (92.8%) of biopsy samples: 15 patients (33.3%) were found to have complete ductopenia, 21 (52.4%) severe ductopenia and 3 (7.7%) mild ductopenia. None of patients had clinical signs of hepatic or intestinal graft-versus-host-disease (GVHD).

Summaries and associations of baseline characteristics and laboratory values of pediatric patients who had undergone chelation therapy with DFX are shown in Table 1.

**Table 1:** Baseline characteristics of patients in the study

Baseline characteristics	Whole cohort	Moderate iron overload(100 > LIC < 200 μmol/g)	Severe iron overload(LIC ≥ 200 μmol/g)	*p* Value
**Patients n (%)**	42 (100)	16 (38.1)	26 (61.9)	-
**Sex (male/female)**	27/15	7/9	20/6	0.0470*
**Age, years, median at transplant (range)**	9.7 (2–17)	11.8 (2–17)	8.6 (2–16)	0.0722**
**Underlying disease: n (%)**				
Acute lymphoblastic leukemia	25 (59.5)	9 (36.0)	16 (64.0)	0.7570*
Acute myeloid leukemia	6 (14.3)	4 (66.7)	2 (33.3)	0.1798*
Severe aplastic anemia	4 (9.5)	1 (25.0)	3 (75.0)	1.0000*
Hemoglobinopathy	4 (9.5)	None	4 (9.5)	0.2799*
Myelodysplastic syndrome	2 (4.8)	2 (100)	None	0.1394*
Non-Hodgkin lymphoma	1 (2.4)	None	1 (100)	1.0000*
**Disease stage: n (%)^‡^**				
Early	14 (33.3)	12 (85.7)	2 (14.3)	<0.0001*
Intermediate	9 (21.4)	3 (33.3)	6 (66.6)	0.4585*
Late	10 (23,8)	None	10 (100)	0.0005*
**Myeloablative conditioning: n (%)**				
Busulfan-based	19 (45.2)	8 (42.1)	11 (57.9)	0.7527*
TBI-based	23 (54.8)	8 (34.8)	15 (65.2)	0.7527*
**PRBC units received, mean (±SD)**	39.4±17.6	36.2±12.9	41.8±20.1	0.2807**
**Ferritin (ng/mL) at beginning of DFX, mean (±SD)^§^**	3597.2±3505.5	2441.3±788.4	4070.0±4289.4	0.1865**
**LIC (μmol/g) at beginning of DFX, mean (±SD)**	236.2±59.5	173.4±12.5	274.8±40.5	<0.0001**
**PIC (μmol/g) at beginning of DFX, mean (±SD)**	84.0±84.7	18.0±8.1	124.4±85.4	0.0011**
**SIC (μmol/g) at beginning of DFX, mean (±SD)**	250.9±47.1	172.8±26.3	299.0±73.8	<0.0001**
**BIC (μmol/g) at beginning of DFX, mean (±SD)**	219.5±91.5	120.9±43.6	280.2±50.3	<0.0001**
**Histological grade of LIC, mean (±SD)^†^**	3.0±0.9	2.2±0.4	3.9±0.3	<0.0001**
**Ductopenia n (%)**	39 (92.8)	16 (100)	23 (88.5)	0.2753 *
**Organs with iron overload, mean (range)**	3.1 (2–4)	2.2 (2–3)	3.8 (2–4)	<0.0001**
**Degree of ductopenia: n (%)**				
Ductopenia absent	3 (7.1)	None	3 (11.5)	0.2753*
Mild ductopenia	3 (7.1)	None	3 (11.5)	0.2753*
Severe ductopenia	22 (52.4)	8 (50,0)	14 (53.8)	1.0000*
Total ductopenia	14 (33.3)	8 (50,0)	6 (23.1)	0.0980*

LIC, liver iron overload; TBI, total body irradiation; PRBC unit, packed red blood cell unit; SD, standard deviation; DFX, deferasirox; PIC, pancreas iron concentration; SIC, spleen iron concentration; BIC, bone iron concentration.

^§^ Ferritin normal range 30.0–400.0 ng/mL (male); 13.0–150.0 (female).

‡Disease stage was defined according to a previously published classification. This classification was applied to patients with acute leukemia and MDS only [32].

^†^Histological grade of liver iron overload was defined according to a previously published classification [33].

*Fisher's test. **Mann–Whitney test.

### Deferasirox treatment

Minimum steady-state plasma concentrations (C_trough_) of DFX were measured 24 hours after its administration. Mean DFX C_trough_ of the whole study group was 17.4 mcg/mL (range: 0–95.42 mcg/mL). To evaluate safety and tolerability of DFX, patients were divided into two groups: 21 patients with DFX C_trough_ < 10 mcg/mL and 21 with DFX C_trough_ > 10 mcg/mL (> 20 μmol/L) [14].

The mean age of patients with DFX C_trough_ < 10 mcg/mL was lower than that of patients with DFX C_trough_ > 10 mcg/mL [7.8 vs. 12.5 years (*p* = 0.0186)].

The grade of siderosis was significantly higher in the first group (DFX C_trough_ < 10 mcg/mL). Mean tissue iron concentrations (MTIC) were calculated for each patient from mean MRI-based LIC, pancreas iron concentration (PIC), spleen iron concentration (SIC) and bone iron concentration (BIC) values. The mean MTIC in the DFX C_trough_ < 10 mcg/mL group was 261.9 μmol/g and in the DFX C_trough_ > 10 mcg/mL group 133.4 μmol/g (*p* < 0.0001).

Fewer organs were affected by IO in patients in the DFX C_trough_ > 10 mcg/mL than in those in the DFX C_trough_ < 10 mcg/mL group (2.3 vs. 3.9, respectively; *p* < 0.0001).

All patients (n=21) in the DFX C_trough_ > 10 mcg/mL group had ductopenia diagnosed prior to DFX treatment, which was complete in 47.6% of them and severe in 52.4%. Ductopenia prior treatment was detected in 18 (85.7%) of the 21 patients in the DFX C_trough_ < 10 mcg/mL group. The difference in incidence of severe ductopenia in the two groups was not statistically significant (10 patients vs. 11; 47.6% vs 52.4%). However, there was a difference in incidence of complete ductopenia between the two groups: namely, five patients (23.8%) in the DFX C_trough_ < 10 mcg/mL group versus 10 (47.6%) in the DFX C_trough_ > 10 mcg/mL group.

The mean dose of DFX administered was higher in the DFX C_trough_ < 10 mcg/mL group than in the DFX C_trough_ > 10 mcg/mL group (25.9 mg/kg vs. 19.2 mg/kg; *p* = 0,0003).

Clinical characteristics and treatment variables according to patient group are shown in Table 2.

**Table 2:** Deferasirox treatment exposure

	Study group	DFX C_trough_ < 10 mcg/ml	DFX C_trough_ > 10 mcg/ml	*p* Value
**Patients (%)**	42 (100)	21 (50,0)	21 (50,0)	-
**Gender (M/F)**	27/15	16/5	11/10	0,1971*
**Age (years), mean (±SD)**	9,7 (5,0)	7,8 (5,2)	12,5 (4,1)	0,0186**
**BMI (kg/m^2^), mean (±SD)**	18,0 (3,2)	18,1 (3,5)	18,0 (2,8)	0,9899**
**LBM (kg), mean (±SD)**	29,8 (14,8)	25,9 (15,8)	33,7 (12,9)	0,0314**
**Baseline ferritin (ng/ml), mean (±SD)**	3597,2 (3505,5)	4722,3 (4653,7)	2472,0 (935,9)	0,0681**
**Post-treatment ferritin (ng/ml), mean (±SD)**	2287,7(1666,6)	2188,8(1897,4)	2386,7 (1439,8)	0,2891**
**Organs with IO, mean (±SD)**	3,1 (0,9)	3,9 (0,2)	2,3 (0,5)	<0,0001**
**MTIC (μmol/g), mean (±SD)**	197,6 (72,1)	261,9 (30,3)	133,4 (32,6)	<0,0001**
**Presence of ductopenia (%)**	39 (92,8)	18 (85,7)	21 (100)	0,2317*
**Ductopenia ratio (±SD)**†	0,13 (0,4)	0,4 (0,5)	0,1 (0,0)	0,0013**
**Severe ductopenia (%)**	21 (52,4)	10 (47,6)	11 (52,4)	1,0000*
**Total ductopenia (%)**	15 (33.3)	5 (23,8)	10 (47,6)	0,1971*
**DFX dose (mg/kg/day), mean (±SD)**	22,5(6,0)	25,9 (4,7)	19,2 (5,4)	0,0003**
**Total dose (mg/day), mean (±SD)**	753,6 (395,7)	754,8 (365,9)	752,4 (322,1)	0,4771**
**Treatment before TDM (days), mean (±SD)**	11,2 (1,4)	11,3 (1,6)	11,0 (1,2)	0,6981**
**DFX C_trough_ (mcg/ml), mean (±SD)**	17,4 (22,2)	3,3 (2,3)	32,2 (24,1)	-

DFX, deferasirox; C_trough_, measured concentration at the end of a dosing interval at steady state (taken directly before next administration); BMI, body mass index; LBM, lean body mass; SD, standard deviation; IO, iron overload; MTIC, mean tissue iron concentration; TDM, therapeutic drug monitoring.

†Ductopenia is express as the ratio of the number of interlobular bile ducts and number of portal tracts.

*Fisher's test. **Mann Whitney test.

### Safety profile of deferasirox

Drug-related adverse events occurred in 22 patients (52.4%). Adverse events of Grade 3 or higher occurred in 13 patients, representing 59.1% of all DFX-related adverse events. Most patients presented more than one adverse event simultaneously. Patients in the DFX C_trough_ > 10 mcg/mL group accounted for 90.5% of all adverse events and 100% of major adverse events.

Adverse events led to discontinuation of DFX treatment in 17 patients (77.3% of the 22 patients who experienced adverse events). In the DFX C_trough_ < 10 mcg/mL group, one of three patients who experienced adverse events stopped chelation therapy versus 16 (84.2%) patients in the DFX C_trough_ > 10 mcg/mL group. Moreover, in the DFX C_trough_ > 10 mcg/mL group, three patients needed dose reductions to continue their chelation therapy. In this group the most common adverse effects were: fatigue (73.7%) and decreased appetite (68.4%). The most common organ-specific adverse effects were high bilirubin concentrations (78.9%), high creatinine concentrations (78.9%), anemia (57.9%), thrombocytopenia (52.6%), high alanine aminotransferase concentrations (52.6%), and high blood urea nitrogen (52.6%).

The mean duration of DFX treatment in the whole study group was 88.8 days, the difference between the two groups (135.7 days in the DFX C_trough_ < 10 mcg/mL group vs. 41.8 days in the DFX C_trough_ > 10 mcg/mL group) being significant.

The safety profile of DFX in pediatric patients undergoing allogeneic HSCT is shown in Table 3.

**Table 3:** Safety profile of deferasirox in pediatric patients with systemic siderosis underwent allogeneic HSCT

Adverse event, patients (%)	Study group(42 patients)	DFX C_trough_ < 10 mcg/mL(21 patients)	DFX C_trough_ > 10 mcg/mL(21 patients)	p Value
**Adverse event, n (%)**:				
***Any DFX-related adverse event***	22 (52.4)	3 (14.3)	19 (90.5)	0.0001*
***Any Grade 1–2 adverse event^#^***	9 (40.9)	3 (100)	6 (28.6)	0.4537*
***Any Grade 3–4 adverse event^#^***	13 (59.1)	None	13 (61.9)	0.0001*
***Death (Grade 5)^#^***	None	None	None	None
***Adverse event resulting in discontinuation***	17 (77.3)	1 (33.3)	16 (84.2)	0.0001*
***Dose reduction***	3 (13.6)	None	3 (15.8)	0.2317*
**DFX-related events**:				
***General disorders, n (%)***				
Fatigue	14 (63.6)	None	14 (73.7)	0.0001*
Decreased appetite	14 (63.6)	1 (33.3)	13 (68.4)	0.0002*
Nausea, vomiting	10 (45.5)	1 (33.3)	9 (47.4)	0.0089*
Gastrointestinal pain	9 (40.9)	2 (66.7)	7 (36.8)	0.1300*
Acidosis	3 (13.6)	None	3 (15.8)	0.2317*
***Investigations, mean (±SD)***				
Neutrophils (count/μL)	2453±1623	2993±1563	1914±1532	0.0091**
Hemoglobin (g/dL)	10.5±1.6	11.2±1.2	9.9±1.8	0.0607**
Platelets (x10^3^/μL)	156±102	181±79	132±118	0.0295**
Alanine aminotransferase (U/L)	52.8±56.4	30.0±15.4	75.6±72.1	0.0643**
Aspartate aminotransferase (U/L)	38.9±40.1	25.6±7.7	52.1±53.5	0.0495**
Gamma-glutamyltransferase (U/L)	32.2±32.5	22.9±9.6	41.6±43.5	0.5372**
Blood bilirubin (mg/dL)	0.8±0.6	0.46±0.26	1.09±0.64	0.0005**
Blood direct bilirubin (mg/dL)	0.3±0.2	0.15±0.05	0.40±0.16	<0.0001**
Blood creatinine (mg/dL)	0.7±0.4	0.4±0.3	0.9±0.4	0.0002**
Blood urea nitrogen (mg/dL)	35.8±17.5	25.7±9.9	45.9±17.8	0.0002**
**DFX treatment duration (days), mean (±SD)**	88.8±82.9	135.8±56.8	41.8±79	<0.0001**

^#^Adverse events were graded according to the National Cancer Institute's Common Terminology Criteria for adverse events, version 4.02.

HSCT, hematopoietic stem cell transplantation; DFX, deferasirox.

*Fisher's test. **Mann–Whitney test.

Table 4 shows Spearman correlation between DFX C_trough_, ductopenia (expressed as ratio of number of interlobular bile ducts to number of portal tracts) and MTIC versus clinical characteristics of study patients. Statistically significant correlations (positive or negative) were identified between the duration of the DFX treatment versus MTIC (r = 0.6954; *p* < 0.0001) and MTIC versus all laboratory tests performed (*p* < 0.005) except for ferritin (r = 0.2762; *p* = 0.0766).

**Table 4:** Spearman correlation between DFX Ctrough, ductopenia ratio, and MTIC versus clinical variables

Patients, n = 42	DFX C_trough_,μg/mL		Ductopenia,ratio†		MTIC,μmol/g	
	r	p	r	p	r	p
**DFX treatment duration (days)**	−0.6932	<0.0001	0.4299	0.0044	0.6954	<0.0001
**DFX Ctrough (μg/mL)**	-	-	−0.4301	0.0044	−0.9159	<0.0001
**Ferritin (ng/mL)**	−0.1787	0.2573	−0.2334	0.1369	0.2762	0.0766
**Organs with IO (n)**	0.0900	0.5706	−0.4059	0.0076	0.9264	<0.0001
**MTIC (μmol/g)**	−0.9159	<0.0001	0.4649	0.0019	-	-
**Neutrophil (count/μL)**	−0.4203	0.0056	0.3451	0.0252	0.4345	0.0040
**Hemoglobin (g/dL)**	−0.3031	0.0510	0.2801	0.0723	0.3216	0.0378
**Platelets (×10^3^/μL)**	−0.3680	0.0165	0.2340	0.1357	0.3296	0.0330
**AST (U/L)**	0.4412	0.0034	−0.4725	0.0016	−0.4168	0.0060
**ALT (U/L)**	0.4005	0.0086	−0.3660	0.0171	−0.3869	0.0114
**Blood total bilirubin (mg/dL)**	0.5960	<0.0001	−0.3532	0.0217	−0.3768	0.0139
**Blood direct bilirubin (mg/dL)**	0.7433	<0.0001	−0.4707	0.0016	−0.5231	0.0004
**Clearance creatinine (mL/min/m^2^)**	−0.7196	<0.0001	0.5223	0.0004	0.6729	<0.0001

DFX, deferasirox; MTIC, mean tissue iron concentration; IO, iron overload; AST, aspartate aminotransferase; ALT, alanine aminotransferase.

†Ductopenia is expressed as the ratio of the number of interlobular bile ducts to number of portal tracts.

## DISCUSSION

DFX, a potent and highly selective oral iron chelator, was approved in the USA at the end of 2004 and in Europe in 2006. DFX is licensed for use as first-line therapy for chronic blood transfusion-related iron overload in patients aged 2 years and older [15]. The effectiveness of chelation therapy depends on continually adding adequate amounts of the chelator to the body iron pool. Because it has a relatively long half-life of 11–19 h, administration of daily doses of 20 mg/kg DFX achieves steady-state trough concentrations of approximately 20 μmol/L [16].

An extensive clinical trial involving more than 1900 patients has demonstrated that deferasirox 20–30 mg/kg/day reduces or maintains iron burden in adult and pediatric patients with a variety of transfusion-dependent anaemias, including β-thalassemia, sickle cell disease, aplastic anemia, myelodysplastic syndrome, and miscellaneous rare anemias [17]. Pharmacokinetic studies on patients receiving chelation therapy have demonstrated that mean plasma trough concentrations of DFX in the steady-state of 20 μmol/L (7.46 mcg/mL) [14]. Only one study has evaluated the safety and tolerability of deferasirox in pediatric patients with transfusion-dependent β-thalassemia major. These patients received a mean DFX dose of 11.3 mg/kg/day and achieved steady-state plasma C_max_ concentrations of 33.5 μmol/l (12.5 mcg/mL) and 40.1 μmol/L (14.9 mcg/mL) in children and adolescents respectively [18]. C_trough_ is about 25% of peak concentrations (C_max_) [19].

The most frequently reported adverse effects in pediatric and adult patients with transfusion-dependent anaemias are generally mild. They include transient gastrointestinal events in 15%, skin rashes in 11%, and mild, dose-dependent increases in serum creatinine in 38% of patients [15]. Neutropenia, agranulocytosis, worsening anemia, and thrombocytopenia, including fatal events, have been reported, mostly in the patients with pre-existing hematologic disorders [20].

Current knowledge about the clinical effects, safety, and tolerability of DFX in pediatric and adult patients with transfusion-dependent anaemias is based on clinical studies conducted on extremely large cohorts. In contrast, there are few data on tolerability and safety of DFX in HSCT recipients.

The most representative research was a multicentric prospective study conducted on 76 adult recipients of allo-HSCT who underwent DFX chelation treatment [21]. Another two studies involved 43 and 23 adult patients who underwent DFX chelation following HSCT [22, 23].

There are almost no data on safety and tolerability of DFX treatment in pediatric recipients of allo-HSCT. The most important pediatric group studied consisted of 12 β-thalassemia major patients with IO aged from 2 to 18 years who had undergone HSCT [24]. Jaekel et al. documented drug-related adverse events in 54 patients (71.1%), 61% of which were mild and 36% moderate in severity. The most common drug-related adverse effect was increase in creatinine concentrations (26.5%); thrombocytopenia (5.5%) and neutropenia were also reported (2.3%). Reductions in DFX dose or temporary interruption because of adverse events were required in 65 patients (85.5%) [21]. Apart from a significant higher number of dose-reductions or interruptions of treatment for severe adverse events, these data are comparable to those reported for patients with transfusion-dependent anemias. There are no published data on DFX pharmacokinetics in pediatric transplant recipients. There is no evidence that similar chelation therapy should be administered to patients with transfusion-dependent anemias and to HSTC recipients, the latter being subjected to highly aggressive chemotherapy regimens during their treatment. This point is crucial, particularly for pediatric patients with onco-hematological disorders.

We performed our study to investigate the high incidence of interruption of DFX treatment for treatment-related adverse events. In our institution, DFX treatment is interrupted in 77.3% of patients, resulting in too short a mean treatment duration (88.8 days).

Evaluation of C_trough_ in study patients revealed that a mean C_trough_ of 17.4 mcg/mL (46.6 μmol/L) (range: 0 mcg/mL–95.4 mcg/mL [255, 8 μmol/l]). Concentrations in our cohort were markedly higher than those previously reported (mean 22.5 mg/kg), even though the doses of DFX were comparable.

We excluded any pharmacological interference: all patients had received standard immunosuppressive therapy and the anti-infection prophylaxis recommended for HSCT recipients.

We took into consideration the fact that the main DFX elimination pathway does not function optimally in such patients. Direct and indirect evidence indicates that the main pathway of deferasirox metabolism is via hepatic glucuronidation to metabolites M3 (acyl glucuronide) and M6 (2-O-glucuronide) and excretion is predominantly via the feces (78.5–86.9 % over 7 days) [25]. Unchanged deferasirox in feces most likely includes a small, unabsorbed fraction of the oral dose, deferasirox eliminated via bile or secreted via the gut wall, and deferasirox glucuronides M3 and M6, which are eliminated via the bile and deconjugated in the gut lumen [26].

More than 90% of patients subjected to allo-HSCT in our institution are found to have from mild to complete ductopenia (unpublished personal data). In our study group, 92.8% (39 patients) had ductopenia, the percentage being 100% in the C_trough_ > 10 mcg/mL group. All patients with particularly high C_trough_ (> 25 mcg/mL [67 μmol/L]) were found to have total ductopenia.

We demonstrated a statistically significant inverse correlation between ductopenia ratio and C_trough_ (r = −0.4301, *p* = 0.0044) and positive correlation between ductopenia ratio and duration of DFX treatment (r = 0.4299, *p* = 0.0044).

**Figure 1:**
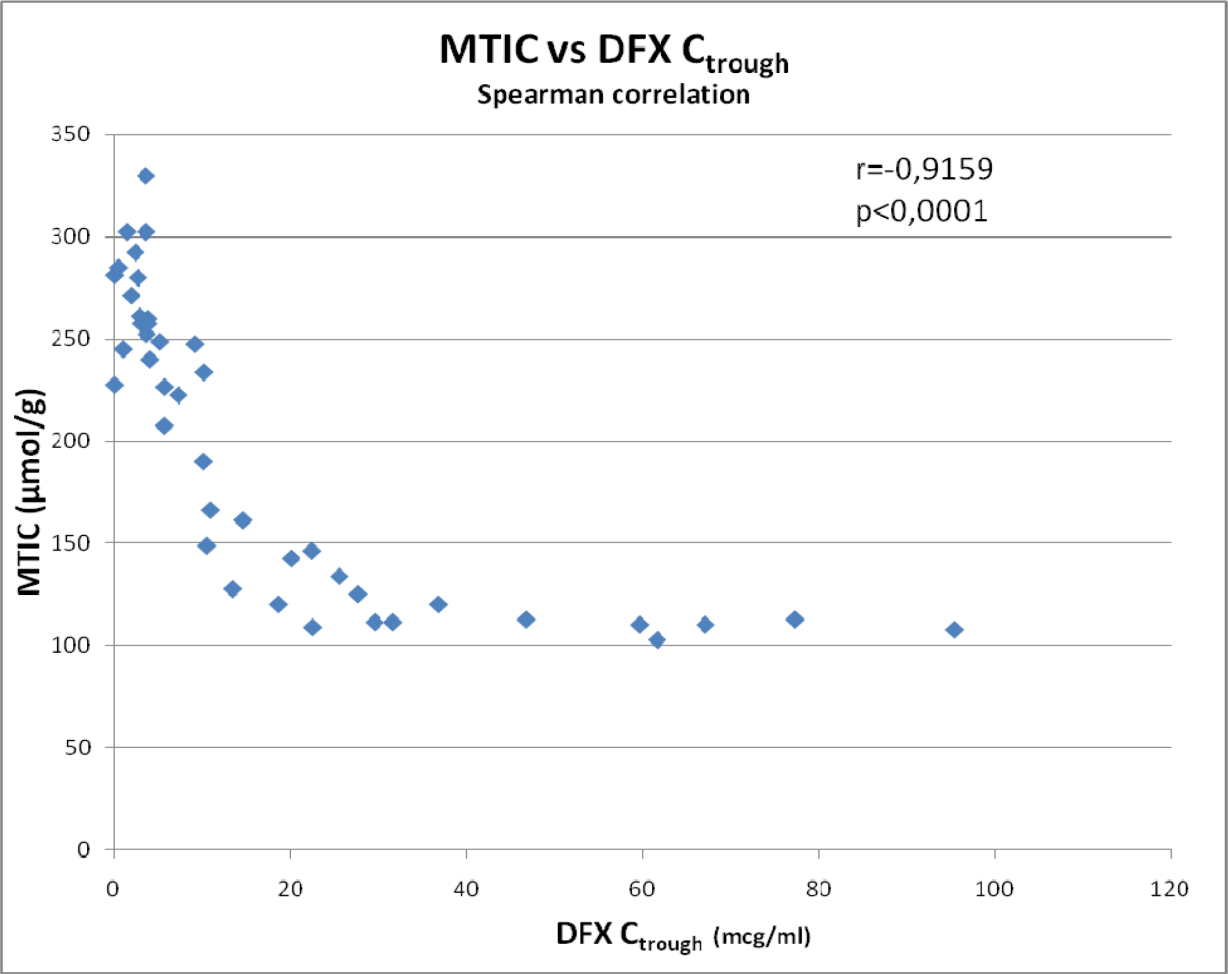
Relationship between mean tissue iron concentration (MTIC) and deferasirox minimum plasma concentration (C_trough_)

Biliary tract injury leading to reduced elimination of the drug is considered to be the main cause of chelator overload. However, a possible secondary cause is the small body iron pool in some patients undergoing allo-HSCT. DFX has a high affinity and specificity for iron. Two DFX molecules are required to form a stable complex with each iron (Fe^3+^) atom [27]. In the C_trough_ > 10 mcg/mL group, the mean tissue iron concentration was half that in the C_trough_ < 10 mcg/mL group (133.4 vs. 261.9 μmol/g, respectively). Our data revealed a significant inverse correlation between MTIC and DFX C_trough_ (r = −0.9159, *p* < 0.0001) and positive correlation between DFX treatment duration and MTIC (r = 0.6954; *p* < 0.0001). Figure 1 shows the strong inverse relationship between MTIC and DFX C_trough_.

We found no significant correlation between MTIC and DFX C_trough_ and serum ferritin concentrations (Table 4). However, it has been well established that high serum ferritin concentrations are not specific for iron overload and do not provide an exact estimation of body iron stores [28, 29]. Serum ferritin has been shown to be only modestly correlated with iron overload in allogeneic HCT recipients [30, 31].

Patients with mild IO and few involved organs likely have fewer iron atoms in parenchymal tissues and therefore fewer iron atoms to bond with DFX molecules. In such patients who also have ductopenia, the excess free DFX molecules cannot be effectively eliminated, leading to accumulation of free DFX molecules in their blood.

In contrast, patients with severe IO likely have more available iron atoms and when too few of them are eliminated, as in patients with mild IO, the circulating DFX consist of both free and chelated components, the latter not being recognized by current detecting methods.

The limitations of this investigation include the retrospective, nonrandomized design and small patient cohort. Because the study was retrospective, we could not perform assess the participants’ genetic characteristic. DFX glucuronidation is controlled by the UDP-glucuronosyltransferase 1A subfamily (UGT1A1 and, to a lesser extent, UGT1A3). UGT1A1 polymorphism has been recognized as an important element in drug tolerance because it increases the risk of toxicity of drugs metabolized via glucuronidation [11].

Pre-transplant evaluation of systemic iron overload allows an early introduction of chelation therapy. This treatment may be particularly useful in preventing the pre-transplant onset or worsening of siderosis in patients subjected to intense chemotherapy regimens for their primary disease.

Based on our results, we would recommend the DFX starting dose of 10 mg/kg/day with close monitoring of pediatric HSCT recipients in order to reduce the incidence of adverse events and the rate of discontinuation of the chelation treatment. DFX treatment (dose: 20–30 mg/kg/day), ordinarily recommended for patients with transfusion-dependent anemias, should be considered in patients without ductopenia only. The sample size of our study is too small: further research is needed to determine the role of ductopenia and of iron overload in the incidence of adverse events during DFX therapy in this specific setting.

## MATERIALS AND METHODS

This single center, retrospective, cohort study was conducted at the Bone Marrow Transplant Unit of Institute for Research in Maternal and Child Health IRCCS Burlo Garofolo in Trieste, Italy. The design and execution of this study were approved by the Ethics Committee of our Institute (Reference no. 1105/2015). Because of the retrospective nature of the study, the requirement to obtain informed consent was waived. Written informed consent for the use of clinical data for research was obtained for all patients at the time of admission to the Transplant Unit.

### Patients

Medical records of all pediatric patients who had undergone allo-SCT in our Institute from January 2011 to June 2016 were retrospectively reviewed and analyzed. Patients without IO, patients with IO who not received any chelation treatment or who had undergone chelation therapy with agents other than DFX, patients who had no plasma samples available for TDM, and those who had not undergone percutaneous liver biopsy were excluded.

The inclusion criteria were as follows: age under 18 years at the time of HSCT, follow-up of survivors for more than six months, presence of IO documented by magnetic resonance imaging (MRI) quantification of liver iron concentration (LIC) performed before transplantation, chelation treatment in post-transplant period with DFX, histological evaluation of hepatic tissue performed after HSCT for diagnostic reasons, and plasma sample(s) collected during DFX treatment and stored at −80°C available for TDM. Application of the exclusion and inclusion criteria resulted in a study cohort comprising 42 patients. Baseline characteristics of these patients are listed in Table 1.

### Chelation treatment

All patients in the study group had from moderate to severe LIC and had undergone oral chelation therapy with deferasirox (Exjade, Novartis, Basel, Switzerland). This treatment begun after discharge from the Transplant Unit and was performed until LIC values had normalized or DFX-related side effects had been identified. For patients who experienced side effects we have considered either dose reduction or discontinuation as reported in the Summary of product characteristics. Discontinuation of DFX treatment criteria were: hematologic involvement (thrombocytopenia, anemia, neutropenia), hepatic or renal impairment or poor compliance because of nausea and vomiting.

### TDM of DFX plasma concentrations

Deferasirox plasma concentrations (C-DFX) were determined by high performance liquid chromatography (HPLC) assay, as previously described and validated [10, 11]. Plasma samples were processed by liquid–liquid extraction using methanol. Sample analysis (100 mcL) was performed using a 150 × 4.6 mm Alltima C18 Column (Alltech, Casalecchio di Reno BO, Italy) at room temperature. The mobile phase consisted of a mixture of disodium hydrogen phosphate (0.05 M) and tetrabutylammonium hydrogen sulfate (0.01 M)–acetonitrile–methanol (42: 12: 46, v/v/v); the elution being carried out at flow rate of 1.3 mL/minute and DFX monitored at a wavelength of 295 nm. The retention time was about 15 min. Calibration points were constructed using a pool of blank human plasma spiked with DFX at concentrations ranging from 1.25 to 60 mcg/mL and the results analyzed by linear regression. The calibration curves of DFX were linear from 1.25 to 60 mcg/mL, resulting in a correlation coefficient o r^2^ = 0.999.

### Histological examination of liver tissue

Histological examination was focused on evaluating the degree of ductopenia. Ductopenia is a one of the histological features traditionally linked to the diagnosis of hepatic graft versus host disease [12] and is expressed as the ratio of the number of interlobular bile ducts to number of portal tracts. Mild ductopenia was diagnosed when > 50% of interlobular bile ducts in all portal tracts of the biopsy sample were present, severe ductopenia when ≤ 50% interlobular bile ducts were present, and total ductopenia when no interlobular bile ducts were present. IO in liver specimens was graded as Grade 0 to Grade 4 according to Scheuer's classification by examination of Perls’ histochemically-stained sections [13].

### Statistical analysis

Continuous variables are expressed as mean ± standard deviation (SD) and categorical variables as frequency, absolute value or percentage. Results were compared by non-parametric statistics; Spearman's rank correlation coefficient was used to assess the relationship between all continuous variables and the clinical variable of biliary ductopenia and the Mann–Whitney *U*-test to compare unpaired data in different groups of patients with or without severe ductopenia or different DFX plasma concentrations. Fisher's exact test was performed to assess the strength of association between categorical variables. All statistical tests were two-sided, with p values <0.05 considered to denote statistical significance. Statistical analysis was performed using WinStat software (v.2012.1, R. Fitch).
